# DSC Study of Collagen in Disc Disease

**DOI:** 10.1155/2009/819635

**Published:** 2010-02-09

**Authors:** S. Skrzyński, A. Sionkowska, A. Marciniak

**Affiliations:** ^1^Department of Neurosurgery, Military Institute of the Health Services, Central Clinical Hospital of the Department of National Defence, Szaserów 128, 00-909 Warsaw, Poland; ^2^Biopolymer Research Group, Faculty of Chemistry, Nicolaus Copernicus University, Gagarina 7, 87-100 Torun, Poland

## Abstract

Differential scanning calorimetry (DSC) has been used to estimate the effect of disc disease on the collagen helix-coil transition and morphology for tissue extracted from patients during surgical operation. Forty discs were obtained from patients with degenerative disc disease undergoing surgery for low back pain. The patients were in the age between 20 and 70 years old. The specimens were kept wet during DSC experiment. The data allow the comparison between thermal stability of collagen tissue from healthy patients and from patients suffering from disc disease. In the paper the comparison between thermal helix-coil transition for collagen fibers from patients suffering from disc disease and collagen fibers from healthy organisms has been discussed. The heating rate has an influence on the position on denaturation temperatures of collagen in disc tissues. Higher helix-coil transition temperature of collagen in degenerated disc suggests that additional intermolecular cross linking of collagen fibers occurs. Denaturation temperatures of collagen in degenerated male disc possess smaller values than in female ones. Disc disease induces changes in collagen structure and leads to formation of additional crosslinks between collagen fibers.

## 1. Introduction

Collagen is the main protein of connective tissue. It has great tensile strength, and is the main component of ligaments and tendons. It is responsible for skin elasticity, and its degradation leads to wrinkles that accompany aging. Collagen also fills out the cornea where it is present in crystalline form. The native tropocollagen molecule (molecular mass 300 kDa) consists of three polypeptide chains of about 1000 amino acid residues each, wound round one another to form a triple helix. The atoms in the individual chains are held together with covalent bonds, while the three chains are held in the triple-helical structure by weaker bonds. When the protein is heat-denatured, these weak bonds are broken but the covalent bonds stay intact and the three chains separate from one another and collapse into random coils [1–3].

Collagen fibers in vivo must be stable enough to withstand the disruptive influence of thermal agitation, but capable of assembly and disassembly of the component molecules. In solution, the unfolding temperatures of a wide range of fibrous collagens are within only a few degrees of the animal's body temperature, but when the molecules are aggregated to form fibers, there is an increase in the transition temperature of ~27°C [4, 5]. Stability of the triple helix in collagen depends on hydrogen bonds. Thermal denaturation of collagen depends on water content, pH of environmental medium, and degree of cross-linking [6–10].

Intervertebral disc disease is one of the most common musculoskeletal disorders. A number of environmental and anthropometric risk factors may contribute to it, and recent reports have suggested the importance of genetic factors as well. Studies of the effect of disc disease on the properties of the collagen molecule are rather limited [11–13]. Differential Scanning Calorimetry DSC has been used to prove the differences between the stages of disc degeneration in calorimetric measures. The structural differences between the stages could be also demonstrated by histology [12]. It has been presented that the annulus fibrosus (AF) and nucleus pulposus (NP) show thermodynamically distinct behavior. The thermal denaturation of normal AF and NP is almost identical regarding the main transition temperatures, but completely different in the total calorimetric enthalpy changes. The comparison of calorimetric curves of the thermal denaturation of degenerated and healthy specimens shows significant differences [11].

It was demonstrated in the literature that DSC is an applicable method for the demonstration of thermal consequences of local as well as global conformational changes in the structure of the human intervertebral discs. It comes from the inherent nature of this method that we cannot assign any thermal event to any molecular process directly, but the results suggest that definitive differences exist between the stages of disc degeneration in calorimetric measurements going on either on a local or global level [13].

The diseases of the intervertebral disc such as degenerative disc disease, and scoliosis are both characterized by changes in the extracellular matrix components that will affect the mechanical function of the tissue. The stability of the collagenous components and hence the mechanical integrity of connective tissues such as the disc are dependent on the degree and type of cross-links between the collagen molecules.

This paper records an experiment that investigates calorimetrically the effect of disc disease on the collagen helix-coil transition for tissue extracted from patients during surgical operation. Forty discs were obtained from patients with degenerative disc disease undergoing surgery for low back pain. The patients were in the age between 20 and 70 years old. The specimens were kept wet during DSC experiment. The data allow the comparison between thermal stability of collagen tissue from healthy patients and from patients suffering from disc disease. 

## 2. Materials and Methods

### 2.1. Materials

Forty discs were obtained from patients with degenerative disc disease undergoing surgery for low back pain in Department of Neurosurgery, Military Institute of the Health Services, Central Clinical Hospital of the Department of National Defence, Warsaw, Poland. The routine was approved by Ethical Commission and all participating patients gave their informed consent. The patients were in the age between 20 and 70 years old. Immediately after operation, the specimens were frozen at −70 degrees C. In the frozen state, the specimens were transported to the laboratory. 

Before DSC experiments, the specimens were kept in room temperature for a while to unfroze them. The specimens were kept wet during DSC experiment.

### 2.2. Differential Scanning Calorimetry (DSC)

Tissues were scanned in a computer-controlled Perkin-Elmer DSC-7, fitted with an Intracooler, and running software supplied by the manufacturer was used for the calorimetric measurements. Weighed samples (±0.01 mg) were heated at 5°C per minute and at 0.5°C per minute from −20°C to an appropriate specified temperature using an empty pan as a reference.

## 3. Results and Discussion

**Figure 1:**
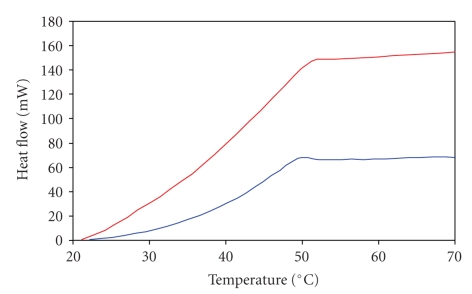
DSC curves of healthy and degenerated disc samples heated with the rate of 0.5°C per minute. (blue line—healthy: women, 36 years old; red line—degenerated disc from woman, 37 years old).

**Figure 2:**
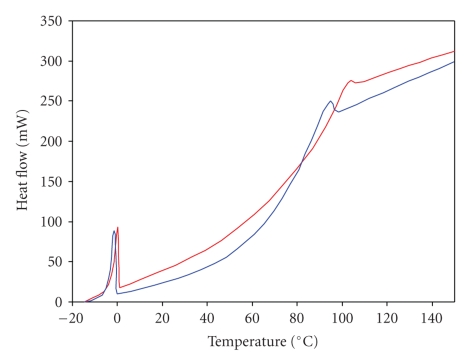
DSC traces of healthy and degenerated disc samples heated with rate of 5°C per minute. (blue line—healthy: women, 36 years old; red line—degenerated disc from woman, 37 years old).

**Figure 3:**
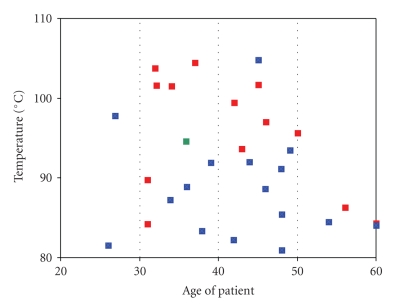
The comparison of denaturation temperatures obtained with heating rate 5°C/min (blue: values for men; red: values for women; green: healthy disc).

DSC curves for collagen in healthy disc and collagen in disc degenerated by discopathy are presented in Figures 1 and 2. The curves in Figure 1 have been obtained with heating rate of 0.5°C/min. The curves in Figure 2 have been obtained with heating rate of 5°C/min. As can be seen the heating rate has an influence on the position of the peak of denaturation temperatures of collagen in disc tissues. The helix-coil transition for collagen from healthy disc appears near 94.5°C (when DSC curves were recorded with heating rate of 5°C/min) and near 50°C (when DSC curves were recorded with heating rate of 0.5°C/min). For collagen in degenerated disc, DSC peak appears in the range of 84–104°C (when DSC curves were recorded with heating rate of 5°C/min) and in the range of 51-52°C (when DSC curves were recorded with heating rate of 0.5°C/min). The data are listed in Tables 1 and 2. DSC profiles show that disc disease induced changes in molecular structure of collagen in disc tissue that alters position in DSC peak of collagen (highly energetic and sharp denaturation endotherm that is a characteristic of the triple helix). Higher helix-coil transition temperature in degenerated disc collagen suggests that additional intermolecular cross-linking of collagen fibers occurs. As a healthy disc came from female disc, we compare DSC curves for healthy and degenerated only for female disc (we did not have male healthy disc). However, we compared DSC curves of female degenerated discs and male degenerated discs. We observed that the maximum of peak responsible for thermal denaturation of collagen in male disc is in the range of 80.95–97.87°C, whereas for female it is in the range of 86.25–104.47°C (Figure 3). It may suggest that collagen in female disc possesses more cross-linking linkages than collagen in male discs.

The high enthalpy of unfolding of collagen is thought to derive mainly from the breaking of the hydrogen bonds between triple helixes and hydrogen bonds forming the hydration network around the collagen molecule. The enthalpy values (ΔH) for the endothermic transition of collagen in disc are shown in Tables 1 and 2. The results show that ΔH decreases with the age of patients and degree of degeneration. The decrease of the enthalpy of this degenerated structure is attributed to the loss of bound water and thermal cooperation of the components. It was also suggested by the widening of the thermal transition period and the asymmetry of the curves themselves. The drop in the main transient temperature in the degenerated disc is mostly due to the loss of the immensely hydrated proteoglycans. The fragmentation of this structure results in the decrease of bound water clusters, and so consequently the decrease of the thermal capacity (ability to store heat energy). In calorimetry, the significantly lower thermal capacity is an important sign of the loss of water clusters, resulting in a greater baseline shift when compared to the native stage. The consequence is the significantly smaller changes in the enthalpy and less thermical cooperation in the degenerated disc in comparison with the control (healthy) specimens.

**Figure 4:**
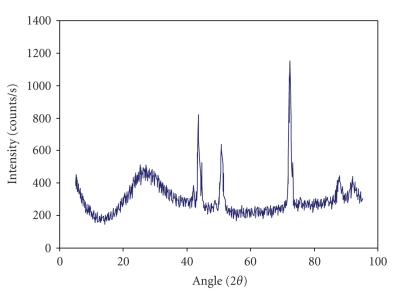
The example of XRD measurement of degenerated disc.

We employed the X-ray diffraction (XRD) analysis to find out whether the crystal domains are present in disc tissue. The examples of XRD spectra are presented in Figure 4. XRD results confirmed the presence of crystal and amorphous phase in collagen. However, we could not find a dependence between the amount of crystal and amorphous phase in collagen and the age of patients nor the gender. 

Disc disease seems to be induced by a process leading to changes in fully hydrated collagen. These changes caused both stabilization and destabilization of the triple helix in fibers. For collagen fibers from patients suffering from disc disease, the cross-linking was observed. The nature of the crosslinks needs deep biochemical studies.

## 4. Conclusions

- The heating rate has an influence on the position on denaturation temperatures of collagen in disc tissues.
- Higher helix-coil transition temperature in degenerated disc collagen suggests that additional intermolecular cross-linking of collagen fibers occurs.
- Denaturation temperatures of collagen in degenerated male disc possess smaller values than in female ones.
- XRD results confirmed the presence of crystal and amorphous phase in collagen.
- Disc disease induces changes in collagen structure and leads to formation of additional crosslinks between collagen fibers.

## Figures and Tables

**Table 1:** Denaturation temperature of disc collagen obtained with the heating rate of 5°C/min.

Patient	Age of patient	Denaturation temperature T (°C)	Enthalpy, ΔH (J/g)
Men	26	81.5	25.4
	27	97.9	30.6
	34	87.4	70.2
	36	88.9	—
	38	83.5	—
	39	92.0	—
	42	82.4	29.9
	44	92.1	14.8
	45	104.8	61.2
	46	88.8	52.7
	48	91.2	66.8
	48	85.5	—
	48	81.0	—
	49	93.5	29.0
	54	84.6	—
	60	84.0	24.0

Women	31	89.8	—
	31	84.2	17.7
	32	101.5	22.6
	32	103.7	18.5
	34	101.5	17,0
	37	104.5	54.9
	42	99.4	33.5
	43	93.7	—
	45	101.7	11.5
	46	97.1	30.5
	50	95.6	61.0
	56	86.3	18.5
	60	84.3	58.3

Healthy control	36	94.6	93.7

**Table 2:** Denaturation temperature of disc collagen obtained with the heating rate of 0.5°C/min.

Patient	Age of patient	Denaturation temperature T (°C)	Enthalpy, ΔH (J/g)
Men	26	51.9	10.6

Women	37	52.1	18.8
	60	51.2	25.8

Healthy control	36	50.3	78.8

## References

[1] Rich A, CricK FH (1961). The molecular structure of collagen. *Journal of Molecular Biology*.

[2] Fraser RDB, MacRea TP, Suzuki E (1979). Chain conformation in the collagen molecule. *Journal of Molecular Biology*.

[3] Bella J, Eaton M, Brodsky B, Berman HM (1994). Crystal and molecular structure of a collagen-like peptide at 1.9 Šresolution. *Science*.

[4] Privalov PL (1982). Stability of proteins which do not present a single co-operative system. *Advances in Protein Chemistry*.

[5] Burjanadze TV (1992). Thermodynamic substantiation of water-bridged collagen structure. *Biopolymers*.

[6] Flory PJ, Garrett RR (1958). Phase transitions in collagen and gelatin systems. *Journal of the American Chemical Society*.

[7] Bigi A, Cojazzi G, Roveri N, Koch MHJ (1987). Differential scanning calorimetry and X-ray diffraction study of tendon collagen thermal denaturation. *International Journal of Biological Macromolecules*.

[8] Luescher M, Ruegg M, Schindler P (1974). Effect of hydration upon the thermal stability of tropocollagen and its dependence on the presence
of neutral salts. *Biopolymers*.

[9] Sionkowska A, Kamińska A (1999). Thermal helix-coil transition in UV irradiated collagen from rat tail tendon. *International Journal of Biological Macromolecules*.

[10] Usha R, Ramasami T (2004). The effects of urea and n-propanol on collagen denaturation: using DSC, circular dichroism and viscosity. *Termochimica Acta*.

[11] Domán I, Tóth Gy, Illés T, Lorinczy D (2001). Differential scanning calorimetric examination of the human intervertebral disc: a preliminary study. *Thermochimica Acta*.

[12] Domán I, Illés T, Lorinczy D (2003). Differential sacnning calorimetric examination of the human intervertebral disc: establishment of calorimetric standards of different stages of degeneration. *Thermochimica Acta*.

[13] Domán I, Illés T (2004). Thermal analysis of the human intervertebral disc. *Journal of Biochemical and Biophysical Methods*.

